# Effect of Facile p-Doping on Electrical and Optoelectronic Characteristics of Ambipolar WSe_2_ Field-Effect Transistors

**DOI:** 10.1186/s11671-019-3137-1

**Published:** 2019-09-12

**Authors:** Junseok Seo, Kyungjune Cho, Woocheol Lee, Jiwon Shin, Jae-Keun Kim, Jaeyoung Kim, Jinsu Pak, Takhee Lee

**Affiliations:** Department of Physics and Astronomy, and Institute of Applied Physics, Seoul National University, Seoul, 08826 Korea

**Keywords:** WSe_2_, Ambipolar field-effect transistors, p-doping, Electrical characteristics, Optoelectronic characteristics

## Abstract

We investigated the electrical and optoelectronic characteristics of ambipolar WSe_2_ field-effect transistors (FETs) via facile p-doping process during the thermal annealing in ambient. Through this annealing, the oxygen molecules were successfully doped into the WSe_2_ surface, which ensured higher p-type conductivity and the shift of the transfer curve to the positive gate voltage direction. Besides, considerably improved photoswitching response characteristics of ambipolar WSe_2_ FETs were achieved by the annealing in ambient. To explore the origin of the changes in electrical and optoelectronic properties, the analyses via X-ray photoelectron, Raman, and photoluminescence spectroscopies were performed. From these analyses, it turned out that WO_3_ layers formed by the annealing in ambient introduced p-doping to ambipolar WSe_2_ FETs, and disorders originated from the WO_3_/WSe_2_ interfaces acted as non-radiative recombination sites, leading to significantly improved photoswitching response time characteristics.

**Electronic supplementary material:**

The online version of this article (10.1186/s11671-019-3137-1) contains supplementary material, which is available to authorized users.

## Background

Two-dimensional (2D) materials have attracted considerable interest as promising candidates for next-generation electronics and optoelectronic devices [1, 2]. Although graphene is one of the most well-studied 2D materials, it lacks an intrinsic bandgap, restricting its wide application. Meanwhile, 2D transition metal dichalcogenides (TMDs), such as MoS_2_, MoSe_2_, WS_2_, and WSe_2_, are advantageous in that they can be used as a channel material of field-effect transistors (FETs) due to their intrinsic bandgap properties, good carrier mobility, and high on/off ratio [2, 3]. Hence, TMDs have been widely used in various devices, such as transistors [4–6], sensors [7–10], logic circuits [11], memory devices [12], field-emission devices [13], and photodetectors [14, 15]. In particular, FETs based on WSe_2_ have demonstrated great ambipolar characteristics such as high carrier mobilities, outstanding photoresponsive properties, excellent mechanical flexibility, and durability [16–18]. Nevertheless, doping WSe_2_ is required to further improve field-effect mobilities or contact properties which are essential in a variety of electronic applications [16, 19]. Among a lot of approaches for doping, thermal annealing in ambient to form WO_3_ layers on a WSe_2_ surface has been demonstrated to be a facile as well as an efficient p-type doping processes [20–22]. For example, Liu et al. thermally annealed WSe_2_ films in ambient without use of additional substances to dope the films in the p-type manner and improved the hole mobility to 83 cm^2^ V^−1^ s^−1^ with employing hexagonal boron nitride substrate [20]. However, thorough studies on the optical and optoelectronic characteristics of WSe_2_ doped by WO_3_ are desired for the optoelectronic applications such as phototransistors, photodiodes, and light-emitting diodes [17, 18, 23, 24].

In this work, we explored the electrical, optical, and optoelectronic properties of ambipolar WSe_2_ FETs before and after thermal annealing in ambient. The oxidized layer (WO_3_) formed on a WSe_2_ surface during the annealing successfully introduced p-doping to the ambipolar WSe_2_ FETs, leading to a shift of the transfer curve to the positive gate voltage direction. Interestingly, long-lasting photoconductivity, which is a phenomenon of the conductance’s being retained after the light irradiation is turned off, disappeared after the annealing. Furthermore, we performed various experiments, such as X-ray photoelectron spectroscopy (XPS), photoluminescence (PL) spectroscopy, and Raman spectroscopy to investigate the origin of the changes in the electrical and photoswitching characteristics of the ambipolar WSe_2_ FETs.

## Methods

WSe_2_ flakes were prepared by the micromechanical exfoliation method from a bulk WSe_2_ crystal, and were transferred to a 270-nm-thick SiO_2_ layer on a heavily doped p++ Si wafer (resistivity ~ 5 × 10^−3^ Ω cm) that was used as the back gate of the FET devices. The thickness of the WSe_2_ flakes was measured using an atomic force microscope (NX 10 AFM, Park Systems). To create electrode patterns, we spin-coated poly(methyl methacrylate) (PMMA) 495K (11% concentration in anisole) as an electron resist layer at 4000 rpm. After the spin-coating, the samples were baked on a hot plate at 180 °C for 90 s. We designed the electrode patterns using an electron-beam lithography instrument (JSM-6510, JEOL), and developed the patterns with a methyl isobutyl ketone/isopropyl alcohol (1:3) solution for 120 s. Finally, titanium metal (30-nm-thick) electrodes were deposited using an electron-beam evaporator (KVE-2004L, Korea Vacuum Tech).

Thermal annealing in ambient was performed on a hot plate at certain temperatures. Thermal annealing in vacuum was performed using a rapid thermal annealing system (KVR-4000, Korea Vacuum Tech) at 4.5 × 10^−4^ Torr and 200 °C for 1 h.

Photoluminescence and Raman spectroscopy measurements were performed using a confocal imaging system (XperRamn 200, Nanobase) with the incident laser wavelength of 532 nm. X-ray photoelectron spectroscopy measurements were performed using an electron energy analyzer (AXIS SUPRA, Kratos). The electrical characteristics of the devices were measured using a probe station (JANIS, ST-500) and a semiconductor parameter analyzer (Keithley 4200-SCS). Photoresponses of the devices were measured under laser (MDE4070V) illumination.

## Results and Discussion

**Fig. 1:**
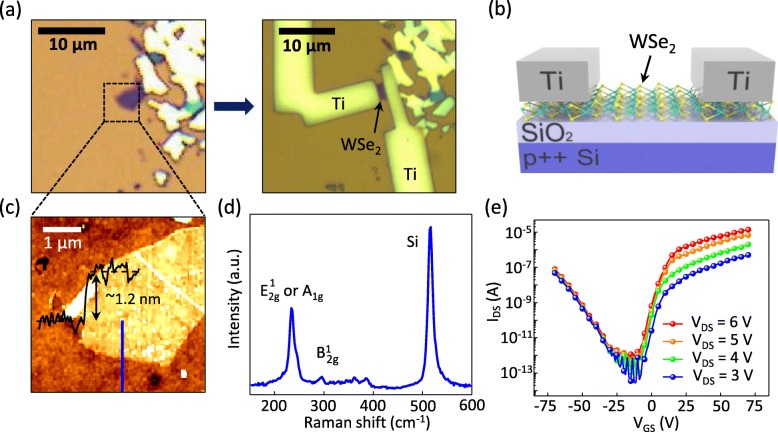
**a** Optical images of a WSe_2_ flake (left) and fabricated WSe_2_ FET (right). **b** Schematic of the fabricated WSe_2_ FET with Ti contacts. **c** AFM image and **d** Raman spectra of WSe_2_. **e** *I*_*DS*_-*V*_*GS*_ curves of the ambipolar WSe_2_ FET

Figure 1a shows the optical images of a WSe_2_ flake and a fabricated WSe_2_ FET. The WSe_2_ flake was mechanically exfoliated from a bulk WSe_2_ crystal and transferred on a 270-nm-thick SiO_2_ surface on a heavily doped p++ Si wafer that was used as the back gate of the FET. Titanium metal patterns used as source and drain electrodes were deposited on the WSe_2_ surface. The detailed device fabrication process is explained in the Additional file 1: Figure S1. A schematic of the fabricated ambipolar WSe_2_ FET is shown in Fig. 1b. All the electrical and photoswitching properties of WSe_2_ FETs were measured in vacuum (~ 3.5 × 10^−3^ Torr) since the oxygen and water molecules in the air can affect the properties of the WSe_2_ FETs. For instance, it has been reported that the semiconducting type of WSe_2_ FETs can be changed from n- to p-type by air exposure [25]. An atomic force microscopy (AFM) image of the WSe_2_ flake is displayed in Fig. 1c with the topographic cross-sectional profile. The measured thickness of the WSe_2_ flake across the blue line was found to be ~ 1.2 nm (an inset graph in Fig. 1c), corresponding to bilayer WSe_2_ (the thickness of a monolayer WSe_2_ is ~ 0.7 nm) [16]. Figure 1d displays the Raman spectrum of a WSe_2_ showing two clear peaks (the peak at 520 cm^−1^ is assigned to the Si substrate). The Raman peak at 245 cm^−1^ corresponds to the in-plane (E^1^_2g_ mode) or out-of-plane (A_1g_ mode) vibrations of WSe_2_, and the Raman peak at 308 cm^−1^ corresponds to the B^1^_2g_ mode that only appears in multilayer WSe_2_ due to the additional interlayer interaction [26]. This finding ensures the good quality of the WSe_2_ flake used in these experiments. The E^1^_2g_ and A_1g_ peaks of WSe_2_ could not be distinguished by the Raman spectroscopy instrument in this study because they are nearly degenerate [27]. Figure 1e shows the transfer curve (source-drain current versus gate voltage; *I*_*DS*_-*V*_*GS*_ curve) of the ambipolar WSe_2_ FET. Such an ambipolar transport behavior of a WSe_2_ FET is due to the number of WSe_2_ layers (bilayer) which can determine the major carrier type in FET [28, 29].

**Fig. 2:**
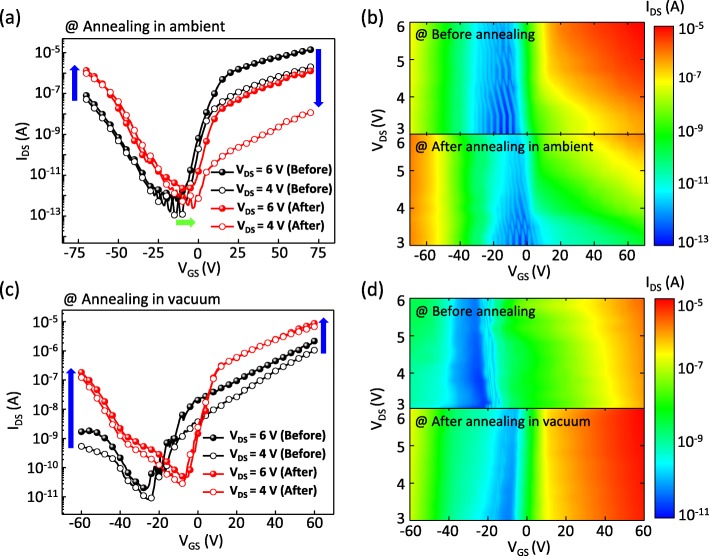
**a**, **c** *I*_*DS*_-*V*_*GS*_ curves on the semilogarithmic scale of a WSe_2_ FET before annealing and after annealing at 200 °C for 1 h. **b**, **d** Contour plots of *I*_*DS*_ as a function of *V*_*GS*_ and *V*_*DS*_ before annealing (upper panel) and after annealing at 200 °C for 1 h (lower panel)

Figure 2a shows the *I*_*DS*_-*V*_*GS*_ curves of the WSe_2_ FET before and after a thermal annealing in ambient at 200 °C for 1 h. The output curves (source-drain current versus source-drain voltage; *I*_*DS*_-*V*_*DS*_ curve) of the same WSe_2_ FET before and after the annealing are shown in the Additional file 1: Figure S2. Several points are noted here. First, the voltage at which the type of the majority carriers changes (*V*_*n*↔*p*_) shifted from − 15 to − 5 V after the annealing in ambient (represented by the green arrow in Fig. 2a). Second, the *I*_*DS*_ increased significantly at the *V*_*GS*_ where the majority carriers are holes (*V*_*GS*_ < *V*_*n*↔*p*_) and decreased at the *V*_*GS*_ where the majority carriers are electrons (*V*_*GS*_ > *V*_*n*↔*p*_) after the annealing (represented by the blue arrows in Fig. 2a). This behavior is attributed to the WO_3_ layer formed by the annealing that introduces p-doping into the WSe_2_ FETs [20]. Third, after the annealing, the hole mobility increased from 0.13 to 1.3 cm^2^ V^−1^ s^−1^, and the electron mobility decreased from 5.5 to 0.69 cm^2^ V^−1^ s^−1^. We used the formula *μ* = (d*I*_*DS*_/d*V*_*GS*_) × [*L*/(*WC*_*i*_*V*_*DS*_)] to calculate the carrier mobility, where *L* (~ 1.5 μm) is the channel length, *W* (~ 2.8 μm) is the channel width, and *C*_*i*_ = *ε*_*0*_*ε*_*r*_ /*d* = 1.3 × 10^−4^ F m^−2^ is the capacitance between WSe_2_ and the p++ Si wafer per unit area. Here, *ε*_*r*_ (~ 3.9) is the dielectric constant of SiO_2_ and *d* (270 nm) is the thickness of the SiO_2_ layer. These changes in the electrical properties after the annealing can be observed more clearly in the contour plots that show the *I*_*DS*_ as a function of *V*_*GS*_ and *V*_*DS*_ before (upper panel) and after (lower panel) the annealing in ambient (Fig. 2b). These contour plots were made based on a lot of *I*_*DS*_-*V*_*GS*_ curves measured in the *V*_*GS*_ range from − 70 to 70 V with a 1.25 V step and *V*_*DS*_ range from 3 to 6 V with a 0.25 V step. The blue regions in the contour plots shifted toward the positive *V*_*GS*_ direction after the annealing. This shift is consistent with the transfer curve shift shown by the green arrow in Fig. 2a. The change in the color at the positive and negative *V*_*GS*_ (Fig. 2b) after the annealing indicates the change in the channel current of the WSe_2_ FET (Fig. 2a). Other WSe_2_ FETs also showed the same change in the electrical properties after annealing in ambient (see Additional file 1: Figures S3 and S4 in the Additional file). Besides, the change of electrical characteristics by the annealing the WSe_2_ FET in vacuum (~ 4.5 × 10^−4^ Torr) at 200 °C for 1 h was investigated (Fig. 2c, d). In contrast with the results of the FET annealed in ambient, the *I*_*DS*_ increased at both *V*_*GS*_ conditions of *V*_*GS*_ > *V*_*n*↔*p*_ and *V*_*GS*_ < *V*_*n*↔*p*_. The increased *I*_*DS*_ obtained by annealing in vacuum is attributed to the improved WSe_2_-Ti contacts without formation of WO_3_ [30]. From the comparison results, it can be anticipated that p-doping was introduced by interaction with the oxygen molecules during the annealing in ambient. The origins of the change in the electrical characteristics are discussed in more detail via the analysis of XPS data afterward.

**Fig. 3:**
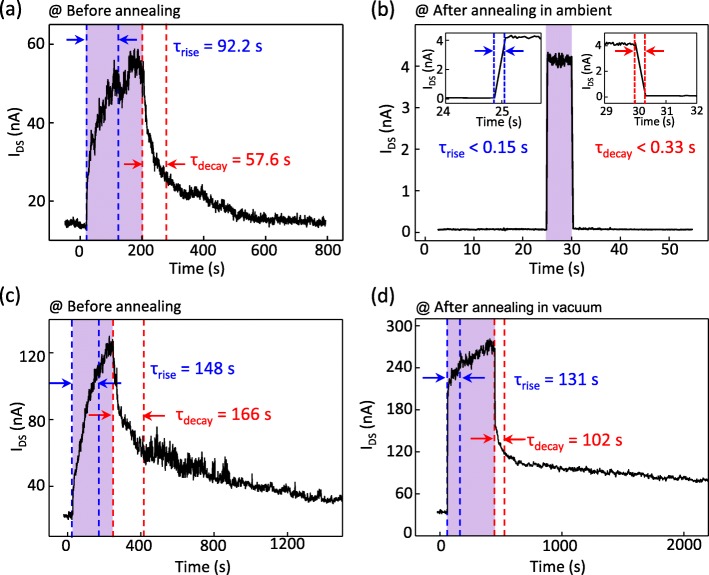
Photoswitching responses of ambipolar WSe_2_ FETs **a**, **c** before and after annealing **b** in ambient at 200 °C for 1 h and **d** in vacuum, respectively. All data were measured at *V*_*GS*_ = 0 V and *V*_*DS*_ = 10 V

Next, we measured the photoswitching characteristics of the WSe_2_ FET before and after the thermal annealing in ambient (Fig. 3a, b). The electrical characteristics of this FET are shown in the Additional file 1: Figure S3. The laser was irradiated onto the WSe_2_ FET and was turned off when the source-drain current appeared to become saturated. Note that the photoswitching experiments were performed at fixed *V*_*GS*_ = 0 V, *V*_*DS*_ = 10 V, the laser wavelength of 405 nm, and the laser power density of 11 mW/cm^2^. Figure 3a, b shows the photoswitching characteristics before and after the annealing in ambient, respectively. In this study, the rise time constant (*τ*_*rise*_) is defined as the time required for the photocurrent (difference between the currents measured in the dark and under irradiation, i.e., *I*_*ph*_ = *I*_*irra*_ – *I*_*dark*_) to change from 10 to 90% of the maximum, and the decay time (*τ*_*decay*_) is the time at which the photocurrent decreases to 1/*e* of its initial value. The purple regions in Fig. 3a, b indicate the time under the laser irradiation. We observed a dramatic change in the photoswitching response times of the WSe_2_ FET after the thermal annealing. Both *τ*_*rise*_ and *τ*_*decay*_ decreased from 92.2 and 57.6 s to less than 0.15 s and 0.33 s, respectively (corresponding to the decrease of more than 610 times and 170 times, respectively). Note that *τ*_*rise*_ and *τ*_*decay*_ after the annealing could not be measured precisely due to instrument limitations. To verify that the change in the photoswitching response times is due to the effect of the oxidation of the WSe_2_ layers, we compared the photoswitching behavior of the WSe_2_ FET before and after thermal annealing in vacuum (~ 4.5 × 10^−4^ Torr) at 200 °C for 1 h (Fig. 3c, d). Contrary to the dramatic decrease of the photoswitching response times for the FET annealed in ambient, a relatively small changes of *τ*_*rise*_ (from 148 to 131 s) and *τ*_*decay*_ (from 166 to 102 s) were observed for the sample annealed in vacuum. This result signifies that the oxidation of the WSe_2_ surface by annealing in ambient is a major origin for the fast photoswitching response. The reason of improved photoswitching behavior by annealing in ambient is that the lattice mismatch between the WSe_2_ and WO_3_ structures provides traps and recombination sites in the bandgap of WSe_2_, which can promote the recombination processes of photogenerated carriers.

**Fig. 4:**
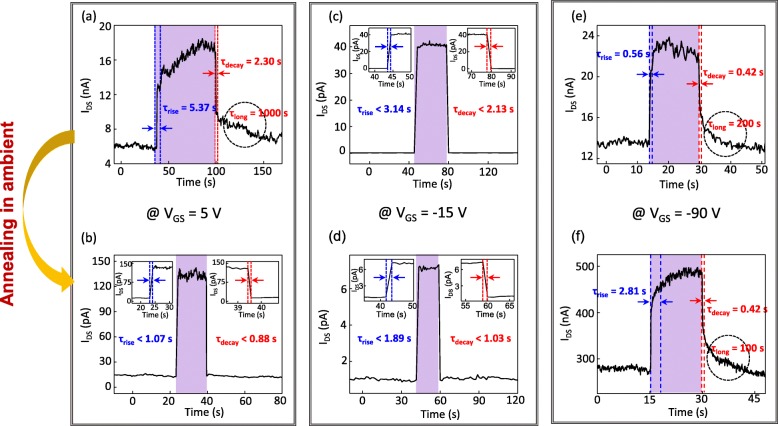
**a** W and **b** Se peaks in XPS spectra of WSe_2_ before and after annealing in ambient at 250 °C for 1 h and 5 h. **c** Schematics of the structural changes in the WSe_2_ caused by thermal annealing in ambient

In addition, for the further investigation on the origin of long-lasting photoswitching characteristics after turning off the laser, the photoswitching characteristics at several *V*_*GS*_ were investigated (Fig. 4). The electrical characteristics of this FET are shown in the Additional file 1: Figure S4. The applied *V*_*GS*_ = 5 V, *V*_*GS*_ = − 15 V, and *V*_*GS*_ = − 90 V correspond to the range of *V*_*GS*_ > *V*_*n*↔*p*_, *V*_*GS*_ ~ *V*_*n*↔*p*_, and *V*_*GS*_ < *V*_*n*↔*p*_, respectively*.* A notable point is that the photoswitching responses strongly relied on the range of *V*_*GS*_ whether it was annealed or not. As decreasing *V*_*GS*_ from 5 to − 90 V in case of before the annealing, the long-lasting photoconductivity (marked as dotted circles in Fig. 4) disappears at *V*_*GS*_ = − 15 V (Fig. 4c) and then reappeared at *V*_*GS*_ = − 90 V (Fig. 4e). This *V*_*GS*_-dependent photoswitching characteristics are mainly due to the changed charge carrier dynamics by the applied *V*_*GS*_ [31]. Depending on the applied *V*_*GS*_ affecting the location of Fermi level (E_F_), the amount of injected carriers after turning off the irradiation can be determined (Additional file 1: Figure S5) [31]. We proposed the band diagrams for explaining these complex *V*_*GS*_-dependent photoswitching characteristics in detail when the irradiation is turned on and off (see the section 4 in Additional file 1).

Figure 4a, b shows that photoswitching characteristics became improved at *V*_*GS*_ = 5 V (*V*_*GS*_ > *V*_*n*↔*p*_) by the thermal annealing, which is in agreement with the results in Fig. 3. This behavior also can be explained by the promoted recombination processes at the induced recombination sites between WSe_2_ and WO_3_ interface. The PL result demonstrated the existence of non-radiative recombination sites at WO_3_/WSe_2_, which will be discussed afterward. At *V*_*GS*_ = − 15 V (*V*_*GS*_ ~ *V*_*n*↔*p*_), we could not observe the distinct change after the thermal annealing due to the highly rapid photoswitching characteristics (Fig. 4c, d). This rapid photoswitching behavior originates from the location of E_F_ in the middle of WSe_2_ bandgap, which suppresses the additional charge injection after turning off the irradiation (see the section 4 in Additional file 1 for detail). For the case of *V*_*GS*_ = − 90 V (Fig. 4e, f), *τ*_*decay*_ and *τ*_*long*_ were maintained and shortened, respectively, although the current after the annealing was much higher than that before the annealing (more than 20 times). Importantly, there is a trade-off between the photo-induced current and decay time constants in phototransistors, because the trapped photogenerated minority carriers can produce an additional electric field, thereby leading to the increased channel current and demanding continuous charge injection even after the irradiation is turned off [32, 33]. In this regard, the preservation of *τ*_*decay*_ and shortened *τ*_*long*_ in spite of the significantly increased photo-induced current signifies the improved photoswitching characteristics by the annealing in ambient as shown in Fig. 4e, f. Regarding *τ*_*rise*_, the location of E_F_ moves to the valence band by p-doping, which causes non-charge neutrality to become stronger due to the decreased hole trap sites where the photogenerated holes can occupy (Additional file 1: Figure S6a). Due to the strong non-charge neutrality, under the irradiation, the more charges are injected for satisfying the charge neutrality. And, photogenerated carriers will undergo more scattering with free carriers while passing through the channel to contribute to the photocurrent, so that *τ*_*rise*_ time can become longer. For that reason, the *τ*_*rise*_ becomes longer at *V*_*GS*_ = − 90 V after thermal annealing as shown in Fig. 4e, f (see the section 4 in Additional file 1 for more detail).

**Fig. 5:**
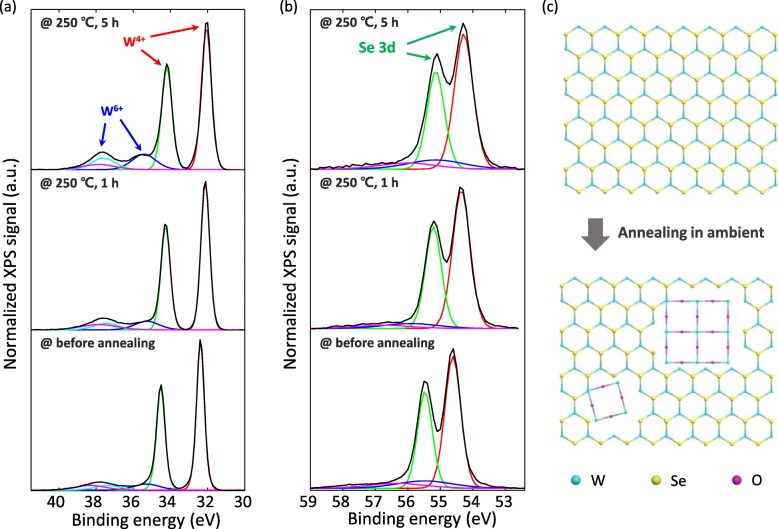
**a** Raman spectra of the WSe_2_ after annealing in ambient at 200 °C for 60 min (black line), at 350 °C for 60 min (red line), and at 500 °C for 5 min (blue line). Inset images correspond to the optical images before and after annealing in 500 °C, respectively. Scale bar = 15 μm. **b** Raman mapping images after annealing at 500 °C integrating with bands at 712 cm^−1^ and 806 cm^−1^, respectively. Scale bar = 10 μm. **c** Optical bandgap of the WSe_2_ before, after annealing in ambient at 250 °C for 30 min, and for 60 min. An inset image is the optical image of a monolayer WSe_2_ flake (labeled as sample 1) with scale bar = 10 μm. **d** Maximum PL intensity and corresponding PL mapping images with a scale bar of 10 μm

Figure 5a, b shows the XPS analyses to investigate the changes in the elemental composition of the WSe_2_ by the thermal annealing in ambient. Although the annealing at 200 °C for 1 h was sufficient to alter both the electrical and photoswitching characteristics as shown in Figs. 2 and 3, these annealing temperature and time were not enough to observe the change in the elemental composition of the WSe_2_. Thus, the mechanically exfoliated WSe_2_ flakes were annealed at 250 °C for 1 h and 5 h in ambient for XPS analyses as shown in Fig. 5a, b. It should be noted that intensities of the two tungsten peaks (labeled as W^6+^ in Fig. 5a) at the binding energies of 35.5 eV and 37.8 eV gradually increased with increased annealing time, whereas no changes were observed in the intensities of the selenium peaks. The tungsten peaks of W^6+^ generated by the thermal annealing indicate the formation of WO_3_ due to the reaction of WSe_2_ with oxygen in air during the annealing [20, 34]. On the other hand, the formation of selenium oxides, such as Se_2_O_3_, was not noticeable (Fig. 5b). Figure 5c exhibits the schematics of microscopic structure before and after WSe_2_ oxidation by annealing, and those are drawn based on the actual geometric structure of WSe_2_ and cubic WO_3_ (W-Se bond length of 2.53 Å, Se-Se bond length of 3.34 Å, and W-O bond length of 1.93 Å) [20, 35, 36]. Since WSe_2_ has a hexagonal structure, while WO_3_ has a cubic structure, the WSe_2_-WO_3_ structure is a quilted in-plane heterojunction, as shown in Fig. 5c [20]. Therefore, the origin of the changed electrical properties after the annealing in ambient (Fig. 2a, b) can be explained by the formation of WO_3_. The formed WO_3_ can serve as an acceptor due to the difference between the work functions of WSe_2_ (~ 4.4 eV) and WO_3_ (~ 6.7 eV) that gives rise to the increased *I*_*DS*_ in the negative *V*_*GS*_ region (*V*_*GS*_ < *V*_*n*↔*p*_) and the decreased *I*_*DS*_ in the positive *V*_*GS*_ region (*V*_*GS*_ > *V*_*n*↔*p*_) [20, 37, 38]. Similar to our results, there have been several reports that a WO_3_ layer which is either deposited on or embedded in a WSe_2_ sheet introduced p-doping into a WSe_2_ FET [20–22].

**Fig. 6:**
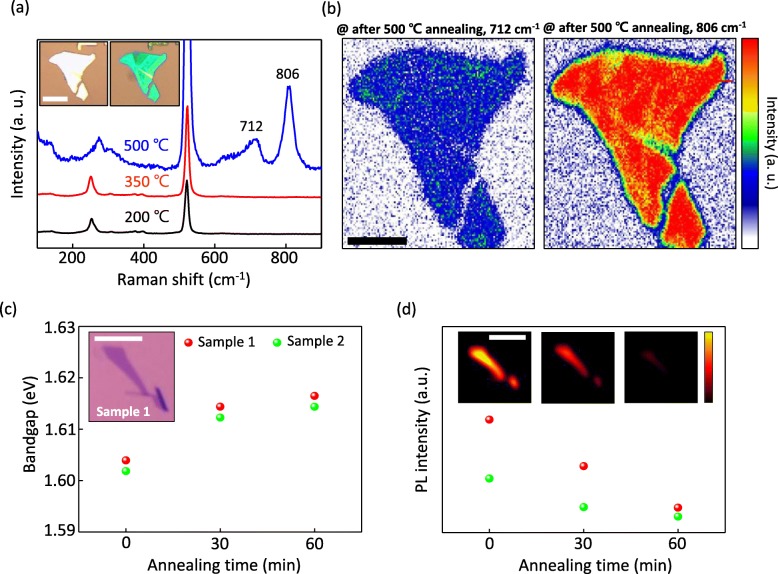
**a** Raman spectra of the WSe_2_ after annealing in ambient at 200 °C for 60 min (black line), at 350 °C for 60 min (red line), and at 500 °C for 5 min (blue line). Inset images correspond to the optical images before and after annealing in 500 °C, respectively. Scale bar = 15 μm. **b** Raman mapping images after annealing at 500 °C integrating with bands at 712 cm^−1^ and 806 cm^−1^, respectively. Scale bar = 10 μm. **c** Optical bandgap of the WSe_2_ before, after annealing in ambient at 250 °C for 30 min, and for 60 min. An inset image is the optical image of a monolayer WSe_2_ flake (labeled as sample 1) with scale bar = 10 μm. **d** Maximum PL intensity and corresponding PL mapping images with a scale bar of 10 μm

We performed Raman and PL spectroscopy experiments to investigate the optical influence by the formation of WO_3_. Figure 6a shows Raman spectra of the WSe_2_ after the annealing in ambient at 200 °C for 60 min (black line), at 350 °C for 60 min (red line), and at 500 °C for 5 min (blue line). The appearance of new peaks around 712 cm^−1^ and 806 cm^−1^ by the annealing at 500 °C, which are very close to the Raman peaks of WO_3_ (709 cm^−1^ and 810 cm^−1^) [39], support the formation of WO_3_ layer on WSe_2_ surface. Inset images are the optical images before and after the annealing at 500 °C for 5 min. Raman mapping images integrating with the bands of 712 cm^−1^ and 806 cm^−1^ in Fig. 6b show the uniform WO_3_ formation on WSe_2_ surface.

PL spectroscopy analysis was conducted for two different monolayer WSe_2_ flakes (labeled as sample 1 and sample 2) as shown in Fig. 6c. The inset of Fig. 6c corresponds to an optical image of sample 1. Each WSe_2_ flakes were annealed for 30 min and 60 min at 250 °C in ambient. The optical and PL mapping images of the other monolayer WSe_2_ flake (labeled as sample 2) are provided in the Additional file 1: Figure S7. As the annealing time increased, the optical bandgaps of the WSe_2_ became wider. The optical bandgap was extracted from the photon energy of the maximum intensity in PL spectrum because that corresponds to the resonance fluorescence originating from the bandgap. While the optical bandgap of the sample 1 was measured as ~ 1.60 eV before the annealing corresponding to the bandgap of monolayer WSe_2_ [27], the bandgap value changed to ~ 1.61 eV after the annealing for 60 min. Although the increase (~ 10 meV) of the optical bandgap is slight, this phenomenon can be explained by the formation of the WSe_2_-WO_3_ in-plane heterojunctions and the dielectric screening effect. Since WO_3_ has a larger bandgap of 2.75 eV compared to WSe_2_ (1.60 eV for a monolayer) [40], the optical bandgap of the monolayer WSe_2_ flakes increased through the annealing in ambient. Furthermore, the formation of WO_3_ on WSe_2_ can generate a stronger dielectric screening effect due to the larger dielectric constant of WO_3_ (~ 90) compared to that of WSe_2_ (~ 22) [41, 42]. Consequently, the stronger dielectric screening effect leads to the diminished exciton binding energy and slightly increased the optical bandgap during the thermal annealing [43].

Interestingly, in perspective of the PL intensity, it obviously decreased as the annealing time increased as shown in Fig. 6d. The PL quenching behavior of monolayer WSe_2_ can be easily observed in PL mapping images integrating the PL intensity in peak region, as increasing annealing time (inset of Fig. 6d). A similar phenomenon was observed in the MoS_2_ treated by oxygen plasma [44]. These results can be explained as follows. Since WO_3_ has an indirect bandgap [40], the band structure of WSe_2_ may be partially changed to that with an indirect bandgap, which leads to reduced PL intensity. Additionally, the lattice mismatch between the WSe_2_ and WO_3_ structures provides traps and recombination sites in the bandgap of WSe_2_ that can affect the electrical and optical characteristics of the WSe_2_. For instance, disorder, defects, and sulfur vacancies can produce shallow or deep trap sites in the MoS_2_ layers, giving rise to the recombination process [31, 45]. Therefore, as the annealing time increased, disorder and the defects originating from the lattice mismatch of the WSe_2_-WO_3_ structure lead to non-radiative (Shockley-Read-Hall) recombination [45], and to reduced PL intensity. Collectively, the experimental results of the XPS, Raman, and PL spectroscopies demonstrate the formation of WO_3_ on the WSe_2_ surface by the annealing in ambient, and those are in agreement well with recent researches on the oxidation of 2D materials [20, 46]. Besides, from the analysis of PL spectroscopy, it was supported that non-radiative recombination sites induced by WO_3_ layer could contribute to the improved photoswitching characteristics by promoting the recombination processes.

## Conclusions

In summary, we fabricated ambipolar WSe_2_ FETs and studied the electrical properties and photoswitching responses before and after thermal annealing in ambient. We observed that the WSe_2_ FETs were successfully doped in the p-type manner and that the photoswitching responses became considerably faster after the ambient thermal annealing. The XPS, Raman, and PL studies demonstrated that the WO_3_ layer formed on the WSe_2_ surface can play the roles of a p-doping layer and non-radiative recombination sites to promote faster photoswitching behavior. This study provides a deeper understanding of effects on electrical and optoelectronic characteristics of ambipolar WSe_2_ FETs by the facile p-doping process via the thermal annealing in ambient.

## Additional file

Additional file 1: **Figure S1.** Schematics of fabricating processes of WSe_2_ FET. **Figure S2.** *I*_*DS*_-*V*_*DS*_ curves of the WSe_2_ FET a when positive *V*_*GS*_ applied and b when negative *V*_*GS*_ applied. Filled and open circular symbols correspond to the curves before and after annealing in ambient, respectively. **Figure S3.** a Transfer curves (*I*_*DS*_-*V*_*GS*_) before (black symbols) and after (red symbols) annealing in ambient. An inset image shows the optical images of the fabricated WSe_2_ FET. b Contour plots which show *I*_*DS*_ as a function of *V*_*GS*_ and *V*_*DS*_ before (upper panel) and after (lower panel) annealing in ambient at 200 ^o^C for 1 h. **Figure S4.** a An optical image of a WSe_2_ FET. b An AFM image (left) of the WSe_2_ flake and the topographic cross-sectional profile along the blue line (right). Scale bar: 1 μm. c *I*_*DS*_*-V*_*GS*_ curves of ambipolar WSe_2_ FET before annealing and after annealing in ambient at 200 ^o^C for 1 h. **Figure S5.** Energy band diagrams describing photoswitching dynamics when the irradiation is turned on at a *V*_*GS*_ > *V*_*n↔p*_, b *V*_*GS*_ ~ *V*_*n↔p*_, c *V*_*GS*_ < *V*_*n↔p*_, and after the irradiation is turned off d-f. **Figure S6.** Energy band diagrams before and after p-doping by WO_3_ under the irradiation at a *V*_*GS*_ < *V*_*n↔p*_ and b *V*_*GS*_ > *V*_*n↔p*_. **Figure S7.** a An optical image of a monolayer WSe_2_ flake (Sample 2). b PL mapping images before annealing (left), after annealing in ambient at 250 ^o^C for 30 min (middle) and 60 min (right). (DOCX 2529 kb)

## Data Availability

All data are fully available without restriction.

## Abbreviations

2D: Two-dimensional;
AFM: Atomic force microscopy
FET: Field-effect transistor;
PL: Photoluminescence;
TMDs: Transition metal dichalcogenides;
XPS: X-ray photoelectron spectroscopy;
