# Supplementary material for: Effect of Facile p-Doping on Electrical and Optoelectronic Characteristics of Ambipolar WSe2 Field-Effect Transistors
Source: Nanoscale Res Lett. 2019 Sep 12;14:313. doi: 10.1186/s11671-019-3137-1 (PMC6742682; doi:10.1186/s11671-019-3137-1)
Supplement: Supplementary file 1 — Figure S1. Schematics of fabricating processes of WSe2 FET. Figure S2. IDS-VDS curves of the WSe2 FET a when positive VGS applied and b when negative VGS applied. Filled and open circular symbols correspond to the curves before and after annealing in ambient, respectively. Figure S3. a Transfer curves (IDS-VGS) before (black symbols) and after (red symbols) annealing in ambient. An inset image shows the optical images of the fabricated WSe2 FET. b Contour plots which show IDS as a function of VGS and VDS before (upper panel) and after (lower panel) annealing in ambient at 200 oC for 1 h. Figure S4. a An optical image of a WSe2 FET. b An AFM image (left) of the WSe2 flake and the topographic cross-sectional profile along the blue line (right). Scale bar: 1 μm. c IDS-VGS curves of ambipolar WSe2 FET before annealing and after annealing in ambient at 200 oC for 1 h. Figure S5. Energy band diagrams describing photoswitching dynamics when the irradiation is turned on at a VGS > Vn↔p, b VGS ~ Vn↔p, c VGS < Vn↔p, and after the irradiation is turned off d-f. Figure S6. Energy band diagrams before and after p-doping by WO3 under the irradiation at a VGS < Vn↔p and b VGS > Vn↔p. Figure S7. a An optical image of a monolayer WSe2 flake (Sample 2). b PL mapping images before annealing (left), after annealing in ambient at 250 oC for 30 min (middle) and 60 min (right). (DOCX 2529 kb) [file 11671_2019_3137_MOESM1_ESM.docx]

Additional file 1

**Effect of Facile p-doping on Electrical and Optoelectronic Characteristics of Ambipolar WSe_2_ Field-Effect Transistors**

Junseok Seo, Kyungjune Cho, Woocheol Lee, Jiwon Shin, Jae-Keun Kim, Jaeyoung Kim, Jinsu Pak* and Takhee Lee^*^

Department of Physics and Astronomy, and Institute of Applied Physics, Seoul National University, Seoul 08826, Korea

*Correspondence: jinsu2060@gmail.com; tlee@snu.ac.kr

Table of Contents

1. Device fabrication process
2. *I_DS_*-*V_DS_* and *I_GS_*-*V_DS_* curves
3. Electrical characteristics of WSe_2_ FETs annealed in ambient
4. *V_GS_*-dependent photoswitching characteristics
5. Photoluminescence data

**1. Device fabrication process**

Figure S1 shows a process of WSe_2_ field-effect transistor (FET) device fabrication. First, we prepared 270 nm-thick SiO_2_ layer on a heavily doped p++ Si wafer (resistivity ~5×10^-3^ Ω cm) which was used as a common back gate. And, WSe_2_ flakes were mechanically exfoliated from a bulk WSe_2_ crystal (purchased from SPI Supplies). Then, the WSe_2_ flakes were transferred from a scotch tape to the SiO_2_ surface. After finding locations of the target WSe_2_ flakes using an optical microscope, thickness of the WSe_2_ flakes was measured by an atomic force microscope (NX 10 AFM, Park Systems). Next, we spin-coated poly(methyl methacrylate) (PMMA) 495K (11% concentration in anisole) at 4000 rpm as an electron resist layer. The samples were baked on a hot plate at 180 ^o^C for 90 s after the spin coating. The patterns of the electrodes were made by an electron beam lithography (JSM-6510, JEOL), and the exposed PMMA regions were developed with a methyl isobutyl ketone/isopropyl alcohol (1:3) solution for 120 s. Finally, Ti metal (30 nm-thick) used as the electrodes was deposited with an electron-beam evaporator system (KVE-2004L, Korea Vacuum Tech). The deposited Ti was lifted off by an acetone with lift-off time of 300 s.

**Figure S1.** Schematics of fabricating processes of WSe_2_ FET.

**2. *I_DS_*−*V_DS_* characteristics**

**Figure S2.** *I_DS_*−*V_DS_* curves of the WSe_2_ FET **a** when positive *V_GS_* applied and **b** when negative *V_GS_* applied. Filled and open circular symbols correspond to the curves before and after annealing in ambient, respectively.

**3. Electrical characteristics of WSe_2_ FETs annealed in ambient**

Figure S3a shows the transfer curve (*I_DS_*-*V_GS_*) at fixed *V_DS_* = 3 V indicating the change of electrical characteristics after the annealing in ambient at 200 ^o^C for 1 h. This device was used in studying the change in photoswitching responses after annealing in ambient as shown in Figs. 3a and 3b. Thickness of the flake was determined to be ~6.9 nm, which corresponds to 10 WSe_2_ layers. Figure S3b show the contour plots representing *I_DS_* as a function of *V_GS_* and *V_DS_* before and after annealing in ambient.

**Figure S3.** **a** Transfer curves (*I_DS_*-*V_GS_*) before (black symbols) and after (red symbols) annealing in ambient. An inset image shows the optical images of the fabricated WSe_2_ FET. **b** Contour plots which show *I_DS_* as a function of *V_GS_* and *V_DS_* before (upper panel) and after (lower panel) annealing in ambient at 200 ^o^C for 1 h.

Figure S4 shows the change in electrical characteristics of another WSe_2_ FET by annealing in ambient, which is consistent with the results of the other devices.

**Figure S4.** **a** An optical image of a WSe_2_ FET. **b** An AFM image (left) of the WSe_2_ flake and the topographic cross-sectional profile along the blue line (right). Scale bar: 1 μm. **c** *I_DS_-V_GS_* curves of ambipolar WSe_2_ FET before annealing and after annealing in ambient at 200 ^o^C for 1 h.

**4. *V_GS_*-dependent photoswitching characteristics**

For *V_GS_* > *V_n↔p_* (*V_GS_* = 5 V in Fig. 4a in the manuscript), the Fermi level (E_F_) is in the vicinity of the E_C_ as shown in Fig. S5a. For the moment, we neglect the trapping of the photogenerated electrons because most electron trap sites are already occupied by non-photogenerated electrons (i.e. electrons which flow under the dark condition) due to the E_F_’s location very close to the E_C_, so only the photogenerated holes can be trapped in the hole trap sites under the irradiation (Fig. S5a). Note that there are band tail states in the TMDs conduction band (E_C_) and the valence band (E_V_) owing to intrinsic disorders or structural defects [S1, S2]. After turning off the irradiation, the photogenerated electrons pass through the channel and most of the photogenerated holes are still trapped in trap sites (Fig. S5d). Thus, non-charge neutrality occurs and can demand the additional charge injection until the charge neutrality is satisfied by disappearance of trapped photogenerated holes, which leads to the long-lasting photoconductivity (Figs. 4a and S5d). This effect denoting that the trapped photogenerated minority carriers induce non-charge neutrality, amplification of photo-induced current, and long-lasting photoconductivity has been reported in 2D TMDs-based photodetectors [S3-S6].

As *V_GS_* decreases, the E_F_ moves towards the E_V_, resulting in both photogenerated electrons and photogenerated holes to be occupied in trap sites. In the regime of *V_GS_* ~ *V_n_*_↔_*_p_* (*V_GS_* = -15 V in Fig. 4c in the manuscript), the amount of trapped photogenerated electrons and trapped photogenerated holes becomes comparable (Fig. S5b), so the additional charge injection is not needed for charge neutrality after turning off irradiation, which leads to the fast photoswitching behavior without the long-lasting photoconductivity (Figs. 4c and S5e). For the case of *V_GS_* < *V_n_*_↔_*_p_* (*V_GS_* = -95 V in Fig. 4e in the manuscript), the E_F_ moves closer to the vicinity of E_V_ (Fig. S5c). Here, E_F_ in case of *V_GS_* < *V_n↔p_* (Fig. S5c) cannot be located close to the E_V_ as it is close to the E_C_ for the case of *V_GS_* > *V_n↔p_* (Fig. S5a) due to the stronger n-type characteristics in WSe_2_ FETs. Hence, although the photogenerated electrons and photogenerated holes can fill the trap sites under the irradiation (Fig. S5c), non-charge neutrality is stronger for the case of *V_GS_* > *V_n↔p_* (Fig. S5a) than that in the case of *V_GS_* < *V_n↔p_* (Fig. S5c). Consequently, after turning off the irradiation, more additional carriers can be injected in the case of *V_GS_* > *V_n↔p_* (Fig. S5d) to satisfy charge neutrality than the case of *V_GS_* < *V_n↔p_* (Fig. S5f). This phenomenon leads to the longer lasting photoconductivity at *V_GS_* *=* 5 V than that at *V_GS_* = -90 V, which can be noticed by larger *τ_long_* (decay time for long-lasting photoconductivity) for the case of *V_GS_**=* 5 V (Fig. 4a) than that for the case of *V_GS_* = -90 V (Fig. 4c). Here, *τ_long_* was obtained by fitting the measured long-lasting photoconductivity with an exponential decay curve.

**Figure S5.** Energy band diagrams describing photoswitching dynamics when the irradiation is turned on at **a** *V_GS_* > *V_n↔p_*, **b** *V_GS_* ~ *V_n↔p_*, **c** *V_GS_* < *V_n↔p_*, and after the irradiation is turned off **d**-**f**.

For the detail explanation on *τ_rise_*, it became longer at *V_GS_* = -90 V and shorter at *V_GS_* = 5 V after the annealing in ambient as shown in Fig. 4 in the manuscript. For the case of *V_GS_* < *V_n↔p_*, the location of E_F_ moves to the E_V_ by p-doping, which causes non-charge neutrality to become stronger due to the decreased hole trap sites where the photogenerated holes can occupy (Fig. S6a). Please note that the trapped original holes could not affect the charge neutrality. Thus, after the thermal annealing, more additional holes can be injected continuously to satisfy charge neutrality. Under the irradiation, the more charge injection requires more time to reach the steady state because the injected holes undergo scattering with free carriers while passing through the channel to contribute to the photocurrent. For that reason, the *τ_rise_* becomes longer at *V_GS_* < *V_n↔p_* after thermal annealing, as shown in Figs. 4e and 4f. Furthermore, the non-radiative recombination sites induced by the WO_3_ formation on WSe_2_ surface can decrease the lifetime of charge carriers, leading to the longer time to reach the steady state. Consequently, the effects of the increased amount of charge injections and the induced recombination sites after the annealing in ambient result in the longer *τ_rise_* at *V_GS_* < *V_n↔p_*.

On the other hand, for *V_GS_* > *V_n↔p_*, the location of E_F_ moves toward E_V_ after the annealing in ambient, which leads to weaker non-charge neutrality due to the increased electron trap sites where the photogenerated electrons can occupy as shown in Fig. S6b. Under the irradiation, less additional electrons can be injected by the weaker non-charge neutrality after the annealing in ambient, so the rise time can be faster due to the mechanism explained in the case of *V_GS_* < *V_n↔p_* (Fig. 4b). Even though the induced non-radiative recombination sites by thermal annealing are able to prolong the time to reach steady state, we think the effect of the amount of injected charge carriers is the more dominant.

**Figure S6.** Energy band diagrams before and after p-doping by WO_3_ under the irradiation at **a** *V_GS_* < *V_n↔p_* and **b** *V_GS_* > *V_n↔p_*.

**5. Photoluminescence data**

Figure S7a displays an optical image of another monolayer WSe_2_ flake (labeled as Sample 2) used in the PL experiments. Its PL mapping images before and after annealing in ambient at 250 ^o^C for 30 min and 60 min are illustrated in Fig. S7b. The change of optical bandgap and maximum PL intensity of the WSe_2_ by the thermal annealing are shown in Figs. 5c and 5d, respectively.

**Figure S7.** **a** An optical image of a monolayer WSe_2_ flake (Sample 2). **b** PL mapping images before annealing (left), after annealing in ambient at 250 ^o^C for 30 min (middle) and 60 min (right).**References**

1. Ghatak S, Pal AN, Ghosh A (2011) Nature of electronic states in atomically thin MoS2 field-effect transistors. ACS Nano 5:7707-7712
2. Kim HJ, Kim DH, Jeong CY, Lee JH, Kwon HI (2017) Determination of interface and bulk trap densities in high-mobility p-type WSe_2_ thin-film transistors. IEEE Electron Device Lett 38:481-484
3. Kufer D, Konstantatos G (2015) Highly sensitive, encapsulated MoS_2_ photodetector with gate controllable gain and speed. Nano Lett 15:7307-7313
4. Bartolomeo AD, Genovese L, Foller T, Giubileo F, Luongo G, Croin L, Liang SJ, Ang LK, Schleberger M (2017) Electrical transport and persistent photoconductivity in monolayer MoS_2_ phototransistors. Nanotechnology 28:214002
5. Fang H, Hu W (2017) Photogating in low dimensional photodetectors. Adv Sci 4:1700323
6. Buscema M, Island JO, Groenendijk DJ, Blanter SI, Steele GA, van der Zant HSJ, Castellanos-Gomez A (2015) Photocurrent generation with two-dimensional van der Waals semiconductors. Chem. Sov. Rev. 44:3691-3718
